# Correlation of microbilirubin with total serum bilirubin and transcutaneous bilirubin

**DOI:** 10.1371/journal.pone.0324201

**Published:** 2025-06-11

**Authors:** Nanthida Phattraprayoon, Kamonwan Soonklang, Peeraya Amnucksoradeja, Tanin Pirunnet

**Affiliations:** 1 Princess Srisavangavadhana Faculty of Medicine, Chulabhorn Royal Academy, Bangkok, Thailand; 2 Department of Pediatrics, Phramongkutklao Hospital and Phramongkutklao College of Medicine, Bangkok, Thailand; Shoklo Malaria Research Unit, THAILAND

## Abstract

Jaundice is a common condition in newborns that requires early detection. Screening for neonatal jaundice involves measuring microbilirubin (MB), total serum bilirubin (TSB), and transcutaneous bilirubin (TcB). This study aimed to examine the correlation between bilirubin levels obtained from these three measurements: MB, TSB, and TcB. The cross-sectional study included infants with a gestational age (GA) of 35 weeks or more who met the criteria for jaundice screening or exhibited signs of jaundice. Bilirubin levels were simultaneously measured using MB, TSB, and TcB at both the forehead and sternum. Statistical analysis was performed using Spearman’s rank correlation coefficient and a Bland-Altman plot. The study included 212 newborns, with 876 measurements (219 datasets). The mean GA ± standard deviation (SD) was 38.31 ± 1.14 weeks, the mean birth weight ± SD was 3094.17 ± 382.18 grams, and the mean age at the time of bilirubin measurement ± SD was 59.8 ± 16.3 hours. The mean ± SD of TSB were 10.42 ± 2.75 mg/dL, with a range of 1.75–20.52 mg/dL. Strong correlations were observed between MB and TSB (r = 0.96, p < 0.001), MB and TcB at the forehead (r = 0.85, p < 0.001), and MB and TcB at the sternum (r = 0.88, p < 0.001). TSB showed good correlations with TcB at both the forehead (r = 0.87, p < 0.001) and sternum (r = 0.90, p < 0.001), as well as correlation between TcB measurements at the forehead and sternum (r = 0.91, p < 0.001). The Bland-Altman analysis demonstrated good agreement between paired bilirubin measurements. The areas under the curve (AUC) for MB and TcB at both the forehead and sternum across three TSB thresholds (>10 mg/dL, > 12 mg/dL, and >15 mg/dL) were all above 0.9, with sensitivity and specificity ≥ 80%. These findings suggest that MB and TcB are useful screening tools in routine practice; however, TSB should still be used to confirm results for the clinical management of neonates with jaundice.

**Trial registration:** Thai Clinical Trials Registry TCTR20231101004

## Introduction

Neonatal jaundice is a condition involving elevated bilirubin, referred to as hyperbilirubinemia. It is common among neonates; in fact, it affects over 80% of full-term infants [1–3]. Infants often show signs of jaundice within the first few weeks of life. Physiological jaundice is a common condition in infants and typically poses no serious medical risks, but it can become harmful if it is severe and left untreated. In contrast, jaundice involving severe hyperbilirubinemia can occur in infants with underlying conditions such as ABO incompatibility, Rhesus incompatibility, or glucose-6-phosphate dehydrogenase (G6PD) deficiency [4,5]. In this latter group, the immediate initiation of treatment is crucial to avoid short- and long-term complications. Hyperbilirubinemia can cause acute bilirubin encephalopathy, which is characterized by various symptoms including poor feeding, lethargy, hypotonia or hypertonia, seizures, high-pitched crying, abnormal posture in the form of retrocollis, and opisthotonus [6–8]. Early detection and timely management of unconjugated hyperbilirubinemia are critical to preventing sequelae such as bilirubin-induced neurological dysfunction [9,10]. The onset of neonatal jaundice typically occurs within the first week of life. However, jaundice persisting beyond three weeks, although sometimes considered normal, warrants further investigation. Several methods have been used to screen for neonatal jaundice. Previously, physicians frequently employed Kramer’s method, which involves observing the yellow part of the infant’s skin to estimate bilirubin levels [11]. However, a number of factors influence the estimated results based on this visual assessment including ethnicity [12,13]. If bilirubin levels are underestimated, this increases the risk of delay in administering the appropriate treatment. Measurement of microbilirubin (MB) is another option for estimating bilirubin levels. This approach requires minimal blood to measure bilirubin levels with a neonatal bilirubin analyzer using a direct spectrophotometry technique with a light-emitting diode (LED) as a light source [14,15]. Therefore, MB is used in regions where access to transcutaneous bilirubinometer for measuring transcutaneous bilirubin (TcB) is limited due to their high cost. The MB measurement requires less blood than the conventional approach for measuring total serum bilirubin (TSB) levels. Consequently, MB measurements are used in some developing countries as a screening method for neonatal jaundice. However, there is a lack of evidence on the correlation among MB, TSB, and TcB. Thus, our objective was to assess whether MB and TcB measured at the forehead and sternum provide reliable alternative estimates of bilirubin level that correlate well with TSB.

## Methods

### Study design and subjects

A cross-sectional observational study was conducted between November 1^st^, 2023 and September 6^th^, 2024, focusing on neonates with a gestational age of 35 weeks or more who required jaundice screening or showed clinical signs of jaundice. These infants were from the nursery, neonatal intensive care unit (NICU), sick newborn ward, or outpatient department at Phramongkutklao Hospital in Bangkok, Thailand. Infants needing bilirubin screening or follow-up measurements of bilirubin levels were also included. The study would exclude infants with skin lesions that could affect the measurement site or those in unstable or critical condition.

The data collected included gestational age, chronological age, blood collection time, and bilirubin levels measured via MB, TSB, and TcB. Written parental consent was required for all infants who involved in the study. All enrolled infants underwent MB, TSB, and TcB testing. TcB measurements were taken at both the forehead and sternum, and blood samples for TSB and MB were collected at the same time. Only measurements obtained prior to the initiation of phototherapy were included in this study.

### Total serum bilirubin measurement

In this method, a blood sample was drawn from a vein, as a larger volume of blood was required for the measurement. Approximately 1 mL of blood was transferred into a lithium heparin or clotted blood tube. The specimen was then analyzed in a biochemical laboratory. TSB measurement was processed using a Cobas® 8000 modular analyzer (Rotkreuz, Switzerland). The machine was calibrated according to the manufacturer’s instructions and the hospital’s protocol.

### Microbilirubin measurement

In this method, heparinized microcapillary tubes were used to collect samples for the MB measurement. Blood was obtained via venous sample into a hematocrit tube, requiring less than 70 µL of blood. The tube was centrifuged at 12,000 rpm for 5 minutes, after which the outer surface of the hematocrit tube was cleaned with gauze or oil-blotting paper. The sample was then analyzed using a neonatal bilirubin analyzer. This analyzer employs a direct spectrophotometry technique, using an LED light source with optical filters at 455 nm and 575 nm, and it provides readings in 3 seconds per sample. The detection range is from 0 to 30 mg/dL. In this study, we utilized the NEO-BIL Plus neonatal bilirubin analyzer (Rome, Italy), which was calibrated according to the manufacturer’s instructions.

### Transcutaneous bilirubin measurement

In this study, TcB was measured using a Dräger Jaundice Meter JM-105 (Lübeck, Germany). Following the manufacturer’s instructions, the device was calibrated daily. The transcutaneous bilirubinometer probe was placed in the center of the forehead, and measurements were taken twice, with the values averaged. The probe was then positioned on the sternum, 2 centimeters above an imaginary line between the nipples, and the measurement was performed twice again, with the results averaged.

### Statistical analysis

The correlation between each pair of bilirubin measurements was evaluated using Spearman’s rank correlation coefficient (r). The Bland-Altman method was used to assess agreement between paired measurements, including mean differences (MD) and the calculation of 95% limits of agreement (LoA) [16]. Receiver operating characteristic (ROC) curves [17] and the area under the curve (AUC) were calculated for MB and TcB measurements taken at the forehead and sternum, using TSB cutoff values of >10 mg/dL, > 12 mg/dL, and >15 mg/dL. Sensitivity and specificity [18] were also analyzed. All statistical analyses were performed using Stata/MP version 18 (StataCorp, College Station, TX).

### Ethical approval

The study protocol was approved by the ethics committees of Chulabhorn Royal Academy (002/2566) and Phramongkutklao Hospital (IRBRTA 1153/2566).

## Results

A cross-sectional study examining the correlations between bilirubin levels from different jaundice screening methods was conducted at Phramongkutklao Hospital in Bangkok, Thailand, from November 2023 to September 2024. The study included 212 newborns who met the inclusion criteria, with parental consent provided for their participation. The participants had a gestational age between 35 and 41 weeks, with a mean gestational age ± standard deviation (SD) of 38.31 ± 1.14 weeks. Birth weights ranged from 2080 to 4492 grams, with a mean ± SD of 3094.17 ± 382.18 grams. The age of participants at the time of measurement ranged from 25 to 145 hours, with a mean ± SD of 59.8 ± 16.3 hours. TSB levels ranged from 1.75 to 20.52 mg/dL, with a mean ± SD of 10.42 ± 2.75 mg/dL. Most of the participants were maternal Rh-positive, and the majority were of Asian ethnicity. According to the hospital protocol, blood group and G6PD testing were not routinely conducted for all newborns. It was only performed in cases where the baby was suspected of having pathologic jaundice, undergoing a jaundice workup, or requiring treatment. Table 1 summarizes the characteristics of both mothers and their infants.

**Table 1:** Characteristics of mothers and infants in the study (Participants 212, Datasets 219, Measurement values 876).

Variable	Mean ± SD	Range	n/N (%)
**Maternal characteristic**			
Maternal age (years)	29.8 ± 5.44	17-43	
Maternal blood group O			77/212 (36.3)
O, Rh-positive			77/212 (36.3)
Maternal blood group non-O			135/212 (63.7)
A, Rh-positive			56/212 (26.4)
A, Rh-negative			1/212 (0.5)
B, Rh-positive			63/212 (29.7)
B, Rh-negative			1/212 (0.5)
AB, Rh-positive			14/212 (6.6)
**Baby characteristic**			
Birth weight (grams)	3094.17 ± 382.18	2080-4492	
Female			105/212 (49.5)
Gestational age (weeks)	38.31 ± 1.14	35-41	
35-36^6/7^			16/212 (7.5)
≥ 37			196/212 (92.5)
Baby blood group: group O *			8/31 (25.8)
O, Rh-positive *			8/31 (25.8)
Baby blood group: group non-O*			23/31 (74.2)
A, Rh positive *			5/31 (16.1)
B, Rh positive *			16/31 (51.6)
AB, Rh positive *			2/31 (6.5)
Baby G6PD deficiency *			4/25 (16)
Direct antiglobulin test (DAT): positive *			1/28 (3.6)
Hemoglobin (g/dL), N = 28 *		12.8-25.4	
Hematocrit (%), N = 28 *		35.1-68.3	
Reticulocyte count (%), N = 10 *		2.1-9.6	
Age of bilirubin measurement (hours)	59.8 ± 16.3	25-145	
Bilirubin level (mg/dL)			
Microbilirubin	10.23 ± 2.42	2.3-18.9	
Total serum bilirubin	10.42 ± 2.75	1.75-20.52	
Transcutaneous bilirubin at the forehead	11.27 ± 2.92	1.1-18	
Transcutaneous bilirubin at the sternum	11.08 ± 2.82	2-18.1	
Need phototherapy **			21/212 (9.9)
Need exchange transfusion			0/212 (0)

**Abbreviations:** dL, decilitre; G6PD, Glucose-6-phosphate dehydrogenase; SD, standard deviation.

* Only the participants who were measured these results.

** Clinical neonatal characteristics of participants who underwent phototherapy were provided in S1Table

Each participant had their bilirubin levels measured at different times, ranging from one to two occasions. Seven participants had their bilirubin levels measured two different times, while for 205 participants, only one measurement was taken. Measurements of MB, TSB, and TcB at the forehead and sternum were performed simultaneously, resulting in datasets containing four bilirubin measurements (TSB, MB, TcB at the forehead, and TcB at the sternum). From the 212 participants, 219 datasets were collected, amounting to a total of 876 measurements.

**Fig 1:**
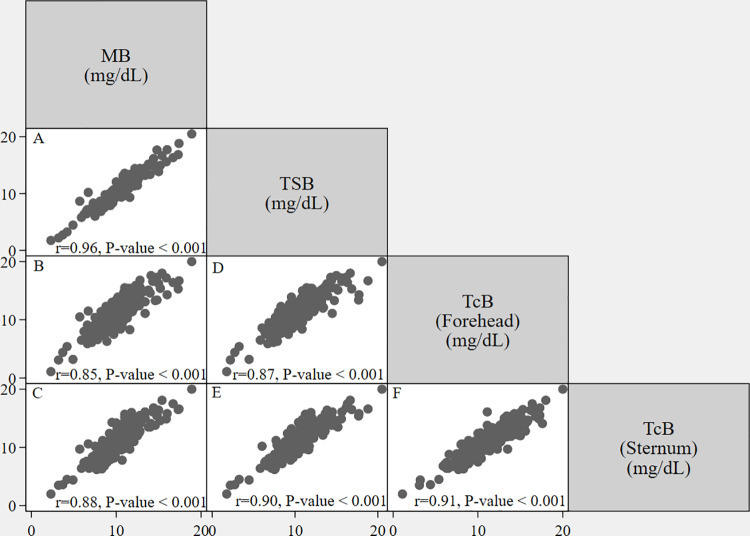
Correlation between bilirubin measurements obtained using different methods or at different anatomical sites before phototherapy (Participants 212, Datasets 219, Measurement values 876). **(A)** The correlation coefficient (r) of MB and TSB. **(B)** The correlation coefficient (r) of MB and TcB measured at the forehead. **(C)** The correlation coefficient (r) of MB and TcB measured at the sternum. **(D)** The correlation coefficient (r) of TSB and TcB measured at the forehead. **(E)** The correlation coefficient (r) of TSB and TcB measured at the sternum. **(F)** The correlation coefficient (r) of TcB measured at the forehead and TcB measured at the sternum. MB, microbilirubin; TcB, transcutaneous bilirubin; TSB, total serum bilirubin.

**Fig 2:**
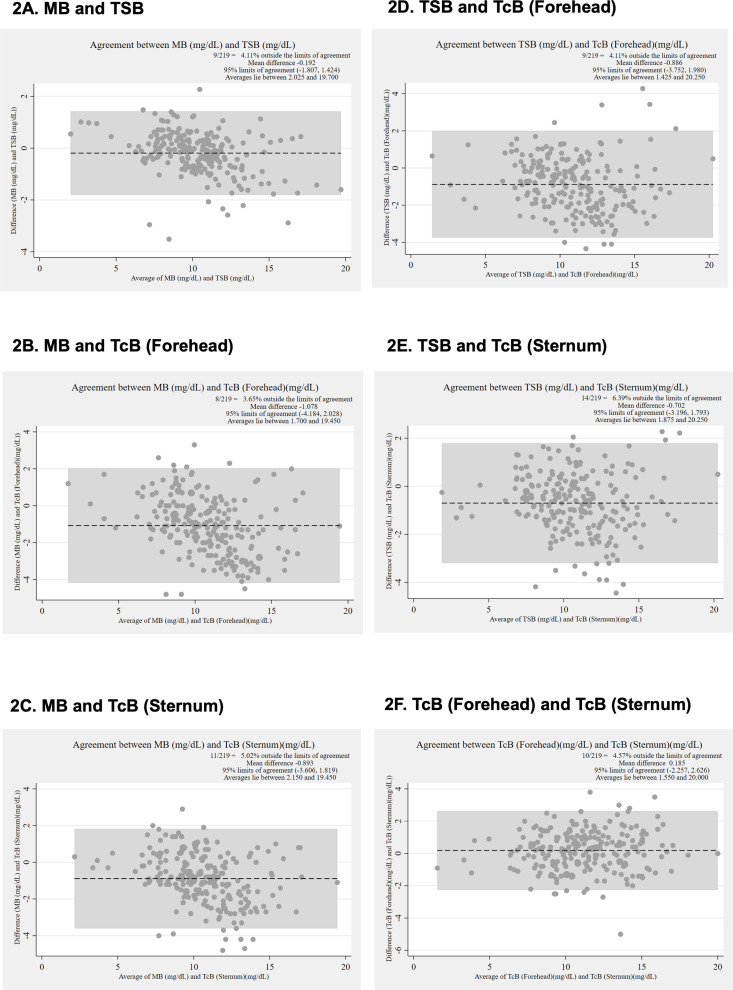
Bland-Altman analysis of bilirubin measurements obtained using different methods or at different anatomical sites before phototherapy (Participants 212, Datasets 219, Measurement values 876). **(A)** Mean difference (MD) and 95% limits of agreement (LoA) between MB and TSB. **(B)** MD and 95% LoA between MB and TcB at the forehead. **(C)** MD and 95% LoA between MB and TcB at the sternum. **(D)** MD and 95% LoA between TSB and TcB at the forehead. **(E)** MD and 95% LoA between TSB and TcB at the sternum. **(F)** MD and 95% LoA between TcB at the forehead and TcB at the sternum. With all values reported in mg/dL. MB, microbilirubin; TcB, transcutaneous bilirubin; TSB, total serum bilirubin.

Strong correlations were observed between MB and TSB (r = 0.96, p < 0.001), MB and TcB at the forehead (r = 0.85, p < 0.001), and MB and TcB at the sternum (r = 0.88, p < 0.001). Additionally, TSB also showed a good correlation with TcB at both the forehead (r = 0.87, p < 0.001) and the sternum (r = 0.90, p < 0.001). TcB measurements taken at the forehead and sternum demonstrated a strong correlation (r = 0.91, p < 0.001). Table 2 and Figs 1 and 2 summarize the correlation coefficients and MDs (95% LoA) and for all comparisons.

**Table 2:** Paired Bilirubin Measurements Using Different Methods or Anatomical Sites Prior to Phototherapy (Participants 212, Datasets 219, Measurement values 876).

Measurement method	Value (mg/dL)	Value range (mg/dL)	MD (95%LoA*)	Correlation coefficient**	P-value**
	Mean ± SD				
**Comparison of MB and TSB**					
MB	10.23 ± 2.42	2.3-18.9	−0.19 (−1.81, 1.42)	0.96	<0.001
TSB	10.42 ± 2.75	1.75-20.52			
**Comparison of MB and TcB at the forehead**					
MB	10.23 ± 2.42	2.3-18.9	−1.08 (−4.18, 2.03)	0.85	<0.001
TcB at the forehead	11.27 ± 2.92	1.1-18			
**Comparison of MB and TcB at the sternum**					
MB	10.23 ± 2.42	2.3-18.9	−0.89 (−3.61, 1.82)	0.88	<0.001
TcB at the sternum	11.08 ± 2.82	2-18.1			
**Comparison of TSB and TcB at the forehead**					
TSB	10.42 ± 2.75	1.75-20.52	−0.89 (−3.75, 1.98)	0.87	<0.001
TcB at the forehead	11.27 ± 2.92	1.1-18			
**Comparison of TSB and TcB at the sternum**					
TSB	10.42 ± 2.75	1.75-20.52	−0.70 (−3.20, 1.79)	0.90	<0.001
TcB at the sternum	11.08 ± 2.82	2-18.1			
**Comparison of TcB at the forehead and TcB at the sternum**					
TcB at the forehead	11.27 ± 2.92	1.1-18	0.19 (−2.26, 2.63)	0.91	<0.001
TcB at the sternum	11.08 ± 2.82	2-18.1			

**Abbreviations:** CI, confidence interval; dL, deciliter; LoA, limit of agreement; MB, microbilirubin; MD, mean difference; mg, milligram; SD, standard deviation; TcB, transcutaneous bilirubin; TSB, total serum bilirubin.

* Using Bland–Altman analysis.

** Using Spearman’s rank correlation coefficient (r) for analysis of the correlation.

The Bland-Altman analysis comparing MB and TSB showed a mean difference of −0.19 (95% LoA −1.81 to 1.42) (Fig 2A). For MB and TcB measured at the forehead, the mean difference was −1.08 (95% LoA −4.18 to 2.03) (Fig 2B). When comparing MB and TcB at the sternum, the mean difference was −0.89 (95% LoA −3.61 to 1.82) (Fig 2C). The analysis of TSB and TcB at the forehead revealed a mean difference of −0.89 (95% LoA −3.75 to 1.98) (Fig 2D), while the difference between TSB and TcB at the sternum was −0.70 (95% LoA −3.20 to 1.79) (Fig 2E). Lastly, the comparison between TcB at the forehead and TcB at the sternum showed a mean difference of 0.19 (95% LoA −2.26 to 2.63) (Fig 2F). The datasets are provided in S2 table.

**Fig 3:**
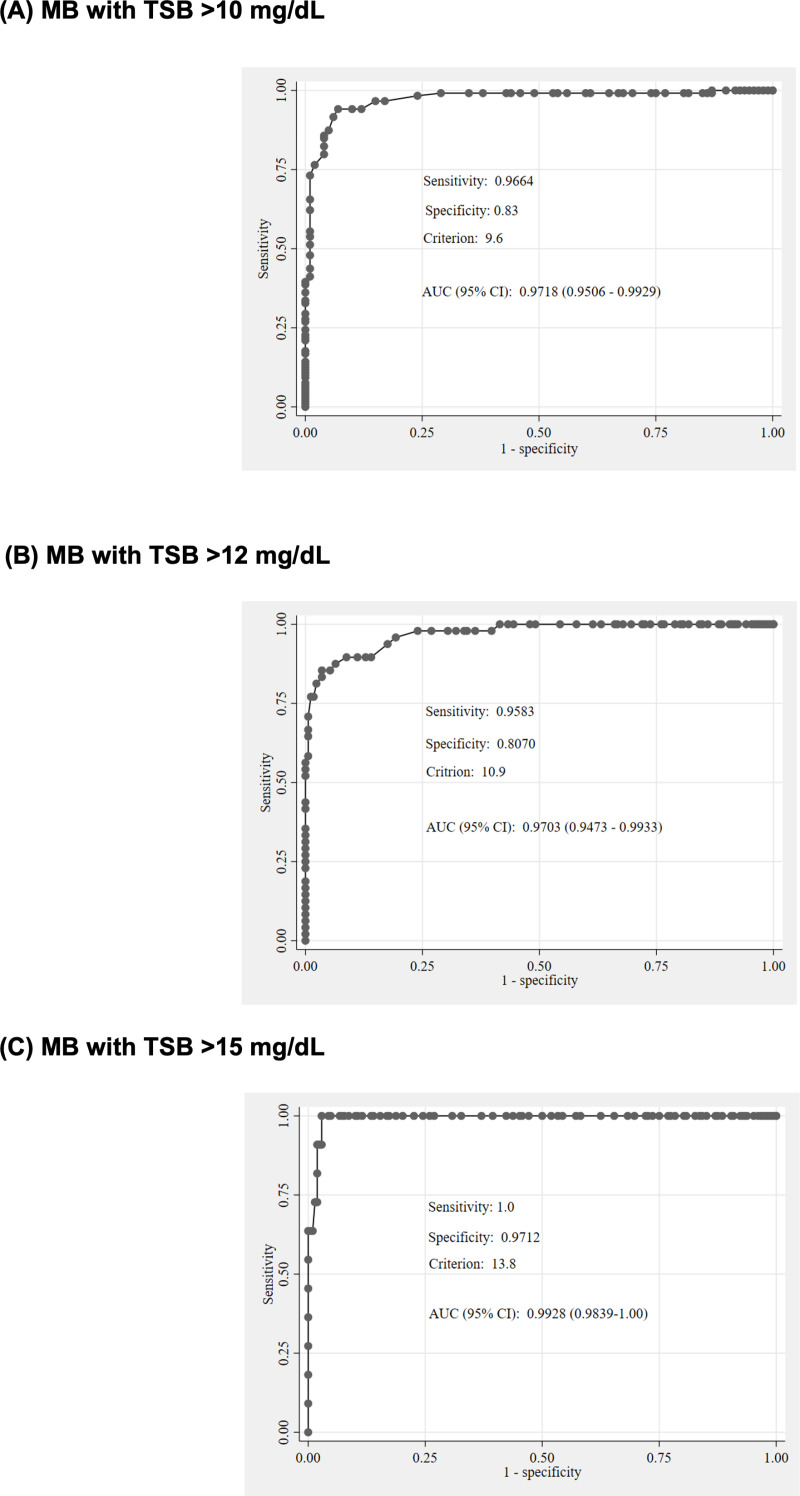
Receiver operating characteristics (ROC) curves for MB (mg/dL) at TSB > 10 mg/dL, > 12 mg/dL, and >15 mg/dL. **(A)** MB with TSB > 10 mg/dL. **(B)** MB with TSB > 12 mg/dL. **(C)** MB with TSB > 15 mg/dL. MB, microbilirubin; TSB, total serum bilirubin.

**Fig 4:**
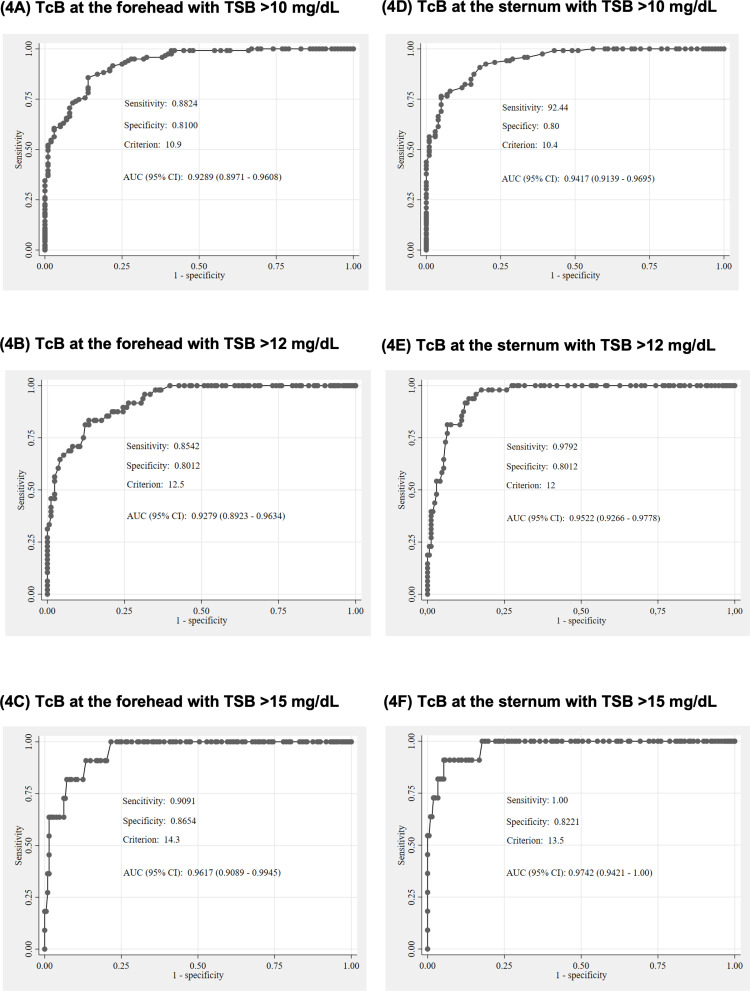
Receiver operating characteristics (ROC) curves for TcB at the forehead, TcB at the sternum (mg/dL) at TSB > 10 mg/dL, > 12 mg/dL, and >15 mg/dL. **(A)** TcB at the forehead with TSB > 10 mg/dL. **(B)** TcB at the forehead with TSB > 12 mg/dL. **(C)** TcB at the forehead with TSB > 15 mg/dL. **(D)** TcB at the sternum with TSB > 10 mg/dL. **(E)** TcB at the sternum with TSB > 12 mg/dL. **(F)** TcB at the sternum with TSB > 15 mg/dL. MB, microbilirubin; TcB, transcutaneous bilirubin; TSB, total serum bilirubin.

We analyzed the ROC curves of MB and TcB measurements at TSB thresholds of >10 mg/dL, > 12 mg/dL, and >15 mg/dL, as shown in Fig 3. The results indicate acceptable sensitivity and specificity for MB in identifying TSB levels at these thresholds. The area under the curve (AUC) for MB was 0.97 at TSB > 10 mg/dL and >12 mg/dL, and 0.99 at TSB > 15 mg/dL (Fig 3A–3C). Similarly, ROC analysis of TcB measured at the forehead showed acceptable diagnostic performance. The AUC for TcB at the forehead was 0.93 at TSB > 10 mg/dL and >12 mg/dL, and 0.96 at TSB > 15 mg/dL (Fig 4A–4C). TcB measurements at the sternum also demonstrated good sensitivity and specificity. The AUC for TcB at the sternum was 0.94 at TSB > 10 mg/dL, 0.95 at TSB > 12 mg/dL, and 0.97 at TSB > 15 mg/dL (Fig 4D–4F).

Out of 219 value, when comparing MB-TSB with a difference of at least ±1 mg/dL, 28 values (12.8%) of MB were underestimated and 14 values (6.4%) of MB were overestimated. For TcB forehead vs. TSB with a difference of at least ±3 mg/dL, 3 values (1.4%) of TcB at the forehead were underestimated and 14 values (6.4%) of TcB at the forehead were overestimated. For TcB at the sternum vs. TSB with a difference of at least ±3 mg/dL, there were no underestimated value, but 11 values (5.0%) of TcB at the sternum were overestimated.

## Discussion

Concerns have been raised about the potential inaccuracies of visual assessments for jaundice [2,19], particularly in infants with darker skin tones [13]. To address these concerns, both non-invasive methods, such as transcutaneous bilirubinometer for TcB, and invasive methods are commonly employed for jaundice screening. However, in settings with limited resources, non-invasive methods like TcB are often less accessible, resulting in the use of invasive techniques, such as measuring MB, as an alternative.

MB requires a smaller blood sample compared to TSB and delivers faster results, making it a practical choice in certain resource-limited settings. This study aimed to evaluate the correlation between bilirubin levels obtained using three different methods: MB, TSB, and TcB at both the forehead and sternum, in near-term and term infants.

While the study demonstrated strong correlations among all methods, with a correlation coefficient of 0.96 between MB and TSB. Additionally, MB showed good correlations with TcB measurements at both the forehead (r = 0.85) and sternum (r = 0.88). However, healthcare providers should be aware that using MB and TcB for screening may result in values that are either underestimates or overestimates compared to those obtained from TSB. Despite these potential discrepancies, the high correlations suggest that MB, along with TcB at the forehead and sternum, could serve as a screening tool for jaundice.

A notable observation was that when comparing MB and TSB results, using a ± 1 mg/dL margin, approximately 12.8% of MB measurements underestimated TSB levels. This discrepancy highlights the importance of confirming MB results with TSB. In contrast, TcB measurements at the forehead and sternum, using a ± 3 mg/dL margin, showed an underestimation of TSB levels in only 0–1.4% of measurements, while overestimation occurred in about 5–6.4% of measurements. This overestimation rate is comparable to that of MB (6.4%). TcB measurement may increase unnecessary blood draws in some cases, while MB measurements might lead to failures in identifying significant hyperbilirubinemia if not confirmed with TSB.

Bland-Altman plots were used to assess the agreement between different bilirubin measurement methods. For MB and TSB, most data points fell within 95% LoA, with only 9 out of 219 (4.11%) lying outside these limits. In contrast, when comparing TSB with TcB at the sternum, 14 out of 219 (6.39%) values were outside the 95% LoA, which was higher than other paired bilirubin measurements. This indicates a slightly lower agreement between TSB and TcB at the sternum compared to MB, TSB, and TcB at the forehead.

In our study, we evaluated the sensitivity and specificity of MB and TcB at the forehead and sternum using different TSB cutoff levels (>10, > 12, and >15 mg/dL). For MB, a cutoff of 9.6 mg/dL yielded a sensitivity of 96.64% and specificity of 83% for detecting TSB > 10 mg/dL. A cutoff of MB 10.9 mg/dL resulted in a sensitivity of 95.83% and specificity of 80.7% for detecting TSB > 12 mg/dL. Notably, using an MB cutoff of 13.8 mg/dL achieved a sensitivity of 100% and specificity of 97.12% for detecting TSB > 15 mg/dL. These findings suggest that when using MB as a screening tool, clinicians should consider using lower values to detect TSB levels.

In contrast, when using TcB at the forehead and sternum to detect TSB levels >10 mg/dL and >12 mg/dL, slightly higher TcB values than the TSB levels were noticed. However, to detect TSB > 15 mg/dL, lower TcB cutoffs at both sites (forehead and sternum) were suggested.

Our findings aligned with those of Akahira-Azuma et al. [20] and Mohamed et al. [21] for TcB measurements at the forehead and sternum, although there was some variation in the cutoff value and criteria. The results are also in agreement with Leite et al., who observed that the direct spectrophotometric method used for MB measurement may yield lower values compared to TSB [22]. Both MB and TcB are valuable screening tools for jaundice, but it is crucial to recognize their limitations. This indicates that while MB may require lower thresholds for effective screening, TcB might necessitate adjustments based on the specific TSB level being targeted.

Therefore, MB and TcB should be used in conjunction with confirmatory tests like TSB, especially when bilirubin levels approach the phototherapy threshold. This approach is consistent with the 2022 AAP guidelines, which recommend TSB confirmation when TcB levels are near or exceed the phototherapy threshold by 3 mg/dL [23]. However, MB measurement lacks clear cut-off points for determining when TSB confirmation is needed, highlighting the need for further research to establish thresholds similar to those defined for TcB. Discrepancies between MB, TcB, and TSB measurements are also possible due to methodological differences. Devices like the NEO-BIL Plus analyzer report a 3% inaccuracy for MB measurements, while the Dräger Jaundice Meter JM-105 provides TcB measurements with an accuracy of ±1.5 mg/dL. Another limitation of MB is that it does not measure direct bilirubin levels, reducing its usefulness in diagnosing conditions related to direct hyperbilirubinemia.

According to 2022 AAP guidelines, TcB or TSB should be measured between 24 and 48 hours after birth, or earlier if discharge occurs sooner, to ensure accurate jaundice screening [23]. Healthcare providers using TcB or MB methods, particularly in low-resource settings, should remain mindful of their limitations. Emerging innovations in jaundice screening, such as point-of-care devices capable of measuring total serum bilirubin with minimal blood requirements, may enhance the accuracy and efficiency of detection while providing immediate results [24,25]. These devices share similarities with MB measurement in terms of simplicity and the need for only a small blood sample.

This study’s strength lies in its investigation of the correlations among three neonatal jaundice screening methods—MB, TcB, and TSB—which, to the best of our knowledge, have not been previously reported together. However, this study has some limitations. First, the measured TSB and MB values ranged from 1.75 to 20.52 mg/dL and 2.3 to 18.9 mg/dL, respectively. This relatively narrow range may limit the applicability of findings to bilirubin levels outside this spectrum. Second, the study primarily included term infants. Therefore, future research should explore correlations at both higher and lower bilirubin levels beyond this range and establish appropriate MB cut-off values requiring TSB confirmation. Further studies are also needed to assess these methods’ correlations in preterm infants.

## Conclusions

This study revealed strong correlations between MB, TSB, and TcB in near-term to term infants. Nonetheless, caution is advised when employing MB as a screening tool, as it may underestimate bilirubin levels, though it is derived from blood samples. The use of TcB should follow AAP guidelines and be confirmed with TSB in specific clinical situations. For effective management of neonatal jaundice in term or near-term infants, TSB confirmation is recommended when using TcB and MB as screening methods.

## Supporting information

S1 Table Clinical neonatal characteristics of participants who underwent phototherapy. (DOCX)

S2 Table The dataset providing the mean and mean difference for each pair, as depicted in the Bland-Altman plots. (DOCX)
