# Supplementary material for: Correlation of microbilirubin with total serum bilirubin and transcutaneous bilirubin
Source: PLoS One. 2025 Jun 11;20(6):e0324201. doi: 10.1371/journal.pone.0324201 (PMC12157342; doi:10.1371/journal.pone.0324201)
Supplement: S1 Table — (DOCX) [file pone.0324201.s001.docx]

**S1 Table.** **Clinical neonatal characteristics of participants who underwent phototherapy**

| **Case** | **Maternal blood group** | **Baby blood group** | **DAT** | **G6PD**  **status** | **% Weight loss before discharge**  **(DOL)** | **Type of feeding** | **Feeding started date**  **(DOL)** | **Diagnosis** |
| --- | --- | --- | --- | --- | --- | --- | --- | --- |
| 1 | O+ | B+ | Neg | Normal | -1.7 %  (DOL 6) | BM/Formula | 1 | ABO incompatibility |
| 2 | B+ | B+ | Neg | Deficiency | -2.7 %  (DOL 4) | BM/Formula | 1 | G6PD deficiency |
| 3 | O+ | B+ | Neg | Normal | -2.5 %  (DOL 4) | BM/Formula | 1 | Unspecified cause |
| 4 | A+ | O+ | Neg | Normal | -1.7%  (DOL 4) | BM/Formula | 1 | Unspecified cause |
| 5 | O+ | O+ | Neg | Normal | -2.9%  (DOL 6) | BM/Formula | 1 | Unspecified cause |
| 6 | AB+ | B+ | Neg | Normal | -2.9%  (DOL 3) | BM/Formula | 1 | Unspecified cause |
| 7 | B+ | B+ | Neg | Normal | +2.7%  (DOL 5) | BM/Formula | 1 | Unspecified cause |
| 8 | A+ | A+ | Neg | Normal | -2.6%  (DOL 4) | BM/Formula | 1 | Unspecified cause |
| 9 | O+ | O+ | Neg | Normal | -2.7%  (DOL 3) | BM/Formula | 1 | Unspecified cause |
| 10 | A+ | A+ | Neg | Normal | 0 %  (DOL 4) | BM/Formula | 1 | Unspecified cause |
| 11 | A+ | A+ | Neg | Normal | -3.7%  (DOL 4) | BM/Formula | 1 | Unspecified cause |
| 12 | A+ | O+ | Neg | Normal | 0 %  (DOL 4) | BM/Formula | 1 | Unspecified cause |
| 13 | B+ | B+ | Neg | Normal | -5.1%  (DOL 4) | BM/Formula | 1 | Most likely suboptimal intake |
| 14 | B+ | B+ | Neg | Normal | -1.7%  (DOL 5) | BM/Formula | 1 | Unspecified cause |
| 15 | B+ | O+ | Neg | Normal | -3.0 %  (DOL 4) | BM/Formula | 1 | Unspecified cause |
| 16 | A+ | O+ | Neg | Normal | -3.8%  (DOL 5) | BM/Formula | 1 | Unspecified cause |
| 17 | A+ | B+ | Neg | Normal | +6.7%  (DOL 4) | BM/Formula | 1 | Unspecified cause |
| 18 | AB+ | B+ | Neg | Deficiency | -4.4%  (DOL 4) | BM/Formula | 1 | G6PD deficiency |
| 19 | O+ | A+ | Neg | Deficiency | +1.7%  (DOL 8) | BM/Formula | 1 | ABO incompatibility  G6PD deficiency |
| 20 | O+ | O+ | Neg | Deficiency | 0 %  (DOL 4) | BM/Formula | 1 | G6PD deficiency |
| 21 | AB+ | B+ | Neg | NR | + 0.4 %  (DOL 10) | Formula | 4 | Early onset sepsis |

**Abbreviations:** BM: breast milk; DAT: Direct antiglobulin test; DOL: Day of life; G6PD: Glucose-6-phosphate dehydrogenase; NR: No report
