# Supplementary material for: Correlation of microbilirubin with total serum bilirubin and transcutaneous bilirubin
Source: PLoS One. 2025 Jun 11;20(6):e0324201. doi: 10.1371/journal.pone.0324201 (PMC12157342; doi:10.1371/journal.pone.0324201)
Supplement: S2 Table — (DOCX) [file pone.0324201.s002.docx]

**S2 Table. The dataset providing the mean and mean difference for each pair, as depicted in the Bland-Altman plots**

| **ID** | **MB-TSB (mg/dL)** | **MB-TcB (Forehead)**  **(mg/dL)** | **MB-TcB (Sternum)**  **(mg/dL)** | **TSB-TcB (Forehead)**  **(mg/dL)** | **TSB-TcB (Sternum)**  **(mg/dL)** | **TcB (Forehead)-TcB (Sternum) (mg/dL)** |
| --- | --- | --- | --- | --- | --- | --- |
| 1 | -0.12 | -3.20 | -4.20 | -3.08 | -4.08 | -1.00 |
| 2 | -1.59 | -3.40 | -2.00 | -1.81 | -0.41 | 1.40 |
| 3 | -0.75 | -0.90 | -1.00 | -0.15 | -0.25 | -0.10 |
| 4 | -1.38 | -1.30 | 0.30 | 0.08 | 1.68 | 1.60 |
| 5 | -1.42 | 0.70 | 0.80 | 2.12 | 2.22 | 0.10 |
| 6 | -2.21 | -3.30 | -1.60 | -1.09 | 0.61 | 1.70 |
| 7 | 1.13 | -1.20 | -1.40 | -2.33 | -2.53 | -0.20 |
| 8 | 0.30 | -1.30 | 1.00 | -1.60 | 0.70 | 2.30 |
| 9 | -1.14 | -3.10 | -2.50 | -1.96 | -1.36 | 0.60 |
| 10 | -0.05 | -0.40 | -0.50 | -0.35 | -0.45 | -0.10 |
| 11 | 0.98 | -0.70 | 0.10 | -1.68 | -0.88 | 0.80 |
| 12 | 1.40 | 1.20 | 1.40 | -0.20 | 0.00 | 0.20 |
| 13 | 0.38 | -0.80 | -2.20 | -1.18 | -2.58 | -1.40 |
| 14 | 0.17 | -1.20 | 0.00 | -1.37 | -0.17 | 1.20 |
| 15 | 0.62 | -0.70 | 0.00 | -1.32 | -0.62 | 0.70 |
| 16 | 0.31 | -0.20 | 0.80 | -0.51 | 0.49 | 1.00 |
| 17 | -1.62 | -1.10 | -1.10 | 0.52 | 0.52 | 0.00 |
| 18 | -0.26 | -3.30 | -4.70 | -3.04 | -4.44 | -1.40 |
| 19 | 0.17 | -2.00 | -0.90 | -2.17 | -1.07 | 1.10 |
| 20 | -0.07 | -2.00 | -1.70 | -1.93 | -1.63 | 0.30 |
| 21 | 0.64 | -3.70 | -3.00 | -4.34 | -3.64 | 0.70 |
| 22 | -0.40 | -4.50 | -3.60 | -4.10 | -3.20 | 0.90 |
| 23 | -0.38 | -3.30 | -2.70 | -2.92 | -2.32 | 0.60 |
| 24 | -1.77 | -2.90 | -2.40 | -1.13 | -0.63 | 0.50 |
| 25 | -1.27 | -2.60 | -2.70 | -1.33 | -1.43 | -0.10 |
| 26 | -1.21 | -3.80 | -2.10 | -2.59 | -0.89 | 1.70 |
| 27 | -2.58 | -4.10 | -4.20 | -1.52 | -1.62 | -0.10 |
| 28 | 0.38 | -0.40 | -0.70 | -0.78 | -1.08 | -0.30 |
| 29 | 0.24 | 1.40 | 1.40 | 1.16 | 1.16 | 0.00 |
| 30 | 0.30 | -3.80 | -0.80 | -4.10 | -1.10 | 3.00 |
| 31 | 0.20 | 0.30 | -1.60 | 0.10 | -1.80 | -1.90 |
| 32 | -0.36 | -1.70 | -0.30 | -1.34 | 0.06 | 1.40 |
| 33 | -0.14 | -1.80 | -1.20 | -1.66 | -1.06 | 0.60 |
| 34 | -0.97 | -1.40 | -1.10 | -0.43 | -0.13 | 0.30 |
| 35 | -0.67 | -1.70 | -1.20 | -1.03 | -0.53 | 0.50 |
| **ID** | **MB-TSB (mg/dL)** | **MB-TcB (Forehead)**  **(mg/dL)** | **MB-TcB (Sternum)**  **(mg/dL)** | **TSB-TcB (Forehead)**  **(mg/dL)** | **TSB-TcB (Sternum)**  **(mg/dL)** | **TcB (Forehead)-TcB (Sternum) (mg/dL)** |
| 36 | 0.73 | -1.00 | -0.90 | -1.73 | -1.63 | 0.10 |
| 37 | 0.17 | 0.20 | -1.60 | 0.03 | -1.77 | -1.80 |
| 38 | 0.24 | 1.20 | -1.00 | 0.96 | -1.24 | -2.20 |
| 39 | -0.31 | 1.00 | 0.20 | 1.31 | 0.51 | -0.80 |
| 40 | 0.16 | -2.80 | -1.40 | -2.96 | -1.56 | 1.40 |
| 41 | -0.48 | -1.00 | -1.30 | -0.52 | -0.82 | -0.30 |
| 42 | 0.76 | 1.10 | 1.40 | 0.34 | 0.64 | 0.30 |
| 43 | 0.62 | 0.80 | -0.60 | 0.18 | -1.22 | -1.40 |
| 44 | -0.08 | -1.10 | -0.80 | -1.02 | -0.72 | 0.30 |
| 45 | -0.90 | -3.50 | 0.00 | -2.60 | 0.90 | 3.50 |
| 46 | -0.99 | -1.10 | -0.80 | -0.11 | 0.19 | 0.30 |
| 47 | -0.43 | 0.30 | 0.20 | 0.73 | 0.63 | -0.10 |
| 48 | 0.55 | -0.10 | -0.50 | -0.65 | -1.05 | -0.40 |
| 49 | 0.26 | -0.40 | -0.10 | -0.66 | -0.36 | 0.30 |
| 50 | -1.46 | -3.60 | -2.40 | -2.14 | -0.94 | 1.20 |
| 51 | -0.95 | -3.00 | -2.60 | -2.05 | -1.65 | 0.40 |
| 52 | -0.72 | -0.90 | -0.50 | -0.18 | 0.22 | 0.40 |
| 53 | -1.62 | -4.00 | -2.50 | -2.38 | -0.88 | 1.50 |
| 54 | 1.03 | 2.60 | 1.50 | 1.57 | 0.47 | -1.10 |
| 55 | 1.34 | -1.30 | 0.10 | -2.64 | -1.24 | 1.40 |
| 56 | -0.01 | -2.70 | -2.40 | -2.69 | -2.39 | 0.30 |
| 57 | -0.92 | -3.50 | -4.80 | -2.58 | -3.88 | -1.30 |
| 58 | 0.95 | -1.20 | -0.30 | -2.15 | -1.25 | 0.90 |
| 59 | -0.82 | -1.00 | -2.10 | -0.18 | -1.28 | -1.10 |
| 60 | -0.17 | -0.90 | -1.50 | -0.73 | -1.33 | -0.60 |
| 61 | -1.73 | -0.43 | -1.20 | 1.30 | 0.53 | -0.77 |
| 62 | -0.40 | -1.30 | -0.90 | -0.90 | -0.50 | 0.40 |
| 63 | -0.49 | -1.90 | -0.40 | -1.41 | 0.09 | 1.50 |
| 64 | -0.31 | -1.10 | 0.50 | -0.79 | 0.81 | 1.60 |
| 65 | -0.68 | -1.60 | -0.50 | -0.92 | 0.18 | 1.10 |
| 66 | -1.05 | -0.80 | 1.00 | 0.25 | 2.05 | 1.80 |
| 67 | -0.15 | 1.30 | 0.80 | 1.45 | 0.95 | -0.50 |
| 68 | 0.60 | -1.50 | 0.30 | -2.10 | -0.30 | 1.80 |
| 69 | 0.55 | 1.20 | 0.30 | 0.65 | -0.25 | -0.90 |
| 70 | -0.39 | 0.50 | 0.40 | 0.89 | 0.79 | -0.10 |
| 71 | 0.06 | -1.00 | -1.10 | -1.06 | -1.16 | -0.10 |
| 72 | -0.49 | -1.20 | -0.20 | -0.71 | 0.29 | 1.00 |
| 73 | -0.35 | 0.50 | 0.30 | 0.85 | 0.65 | -0.20 |
| 74 | -0.12 | -2.90 | -0.60 | -2.78 | -0.48 | 2.30 |
| **ID** | **MB-TSB (mg/dL)** | **MB-TcB (Forehead)**  **(mg/dL)** | **MB-TcB (Sternum)**  **(mg/dL)** | **TSB-TcB (Forehead)**  **(mg/dL)** | **TSB-TcB (Sternum)**  **(mg/dL)** | **TcB (Forehead)-TcB (Sternum) (mg/dL)** |
| 75 | -0.35 | 2.10 | 0.80 | 2.45 | 1.15 | -1.30 |
| 76 | -0.81 | 0.00 | -0.70 | 0.81 | 0.11 | -0.70 |
| 77 | -1.27 | 0.10 | 0.00 | 1.37 | 1.27 | -0.10 |
| 78 | 0.41 | 0.80 | 0.50 | 0.39 | 0.09 | -0.30 |
| 79 | -0.60 | -2.70 | 1.10 | -2.10 | 1.70 | 3.80 |
| 80 | 0.48 | -1.60 | -1.60 | -2.08 | -2.08 | 0.00 |
| 81 | 0.06 | -0.60 | -0.50 | -0.66 | -0.56 | 0.10 |
| 82 | -1.52 | -1.20 | -1.10 | 0.32 | 0.42 | 0.10 |
| 83 | 0.07 | 0.00 | -0.50 | -0.07 | -0.57 | -0.50 |
| 84 | -0.09 | -0.40 | -0.90 | -0.31 | -0.81 | -0.50 |
| 85 | 0.17 | -1.30 | -1.00 | -1.47 | -1.17 | 0.30 |
| 86 | -0.74 | -0.30 | -2.00 | 0.44 | -1.26 | -1.70 |
| 87 | 0.70 | 0.00 | 2.00 | -0.70 | 1.30 | 2.00 |
| 88 | -0.02 | 0.70 | 0.40 | 0.72 | 0.42 | -0.30 |
| 89 | -1.03 | -1.40 | -1.00 | -0.37 | 0.03 | 0.40 |
| 90 | 1.06 | -0.20 | 0.50 | -1.26 | -0.56 | 0.70 |
| 91 | 0.37 | -1.30 | -2.70 | -1.67 | -3.07 | -1.40 |
| 92 | 0.66 | -2.70 | -1.10 | -3.36 | -1.76 | 1.60 |
| 93 | -0.70 | 0.30 | 0.30 | 1.00 | 1.00 | 0.00 |
| 94 | 0.50 | 2.20 | 0.80 | 1.70 | 0.30 | -1.40 |
| 95 | 0.07 | -1.60 | -1.20 | -1.67 | -1.27 | 0.40 |
| 96 | -0.56 | -1.00 | -0.80 | -0.44 | -0.24 | 0.20 |
| 97 | -0.44 | -3.10 | -2.50 | -2.66 | -2.06 | 0.60 |
| 98 | -0.39 | -3.00 | -2.50 | -2.61 | -2.11 | 0.50 |
| 99 | -0.56 | 0.70 | -0.60 | 1.26 | -0.04 | -1.30 |
| 100 | -0.83 | -2.40 | -1.80 | -1.57 | -0.97 | 0.60 |
| 101 | 0.12 | -1.60 | -0.30 | -1.72 | -0.42 | 1.30 |
| 102 | 0.07 | 0.50 | 0.20 | 0.43 | 0.13 | -0.30 |
| 103 | -0.43 | -0.60 | -0.80 | -0.17 | -0.37 | -0.20 |
| 104 | -0.73 | -1.10 | -1.70 | -0.37 | -0.97 | -0.60 |
| 105 | -0.60 | 0.70 | -0.60 | 1.30 | 0.00 | -1.30 |
| 106 | -0.22 | 0.20 | 0.70 | 0.42 | 0.92 | 0.50 |
| 107 | -0.84 | -2.80 | 0.00 | -1.96 | 0.84 | 2.80 |
| 108 | 0.53 | 1.00 | -1.20 | 0.47 | -1.73 | -2.20 |
| 109 | -0.79 | -2.80 | -1.60 | -2.01 | -0.81 | 1.20 |
| 110 | -0.67 | -1.20 | -1.70 | -0.53 | -1.03 | -0.50 |
| 111 | 1.08 | -0.80 | 0.60 | -1.88 | -0.48 | 1.40 |
| 112 | -0.02 | 0.70 | 0.60 | 0.72 | 0.62 | -0.10 |
| 113 | -0.10 | -3.10 | -2.50 | -3.00 | -2.40 | 0.60 |
| **ID** | **MB-TSB (mg/dL)** | **MB-TcB (Forehead)**  **(mg/dL)** | **MB-TcB (Sternum)**  **(mg/dL)** | **TSB-TcB (Forehead)**  **(mg/dL)** | **TSB-TcB (Sternum)**  **(mg/dL)** | **TcB (Forehead)-TcB (Sternum) (mg/dL)** |
| 114 | 1.05 | 1.40 | 1.80 | 0.35 | 0.75 | 0.40 |
| 115 | -0.68 | -0.20 | 0.80 | 0.48 | 1.48 | 1.00 |
| 116 | 0.18 | 0.00 | 1.50 | -0.18 | 1.32 | 1.50 |
| 117 | -0.84 | -1.90 | -1.60 | -1.06 | -0.76 | 0.30 |
| 118 | 0.08 | -2.00 | 0.50 | -2.08 | 0.42 | 2.50 |
| 119 | -0.39 | -0.40 | 0.60 | -0.01 | 0.99 | 1.00 |
| 120 | -3.51 | -4.80 | -3.90 | -1.29 | -0.39 | 0.90 |
| 121 | -2.95 | -4.80 | -4.00 | -1.85 | -1.05 | 0.80 |
| 122 | 0.05 | -2.20 | -1.40 | -2.25 | -1.45 | 0.80 |
| 123 | -0.39 | -0.90 | -2.60 | -0.51 | -2.21 | -1.70 |
| 124 | 0.66 | 1.70 | 1.00 | 1.04 | 0.34 | -0.70 |
| 125 | -0.48 | -1.00 | -3.70 | -0.52 | -3.22 | -2.70 |
| 126 | -0.01 | -0.50 | 0.50 | -0.49 | 0.51 | 1.00 |
| 127 | 1.29 | 1.30 | 0.50 | 0.01 | -0.79 | -0.80 |
| 128 | -0.11 | 0.70 | -0.20 | 0.81 | -0.09 | -0.90 |
| 129 | -0.99 | -1.20 | -1.30 | -0.21 | -0.31 | -0.10 |
| 130 | 0.20 | -2.20 | -0.80 | -2.40 | -1.00 | 1.40 |
| 131 | -0.80 | -2.90 | -1.90 | -2.10 | -1.10 | 1.00 |
| 132 | 0.71 | 0.00 | 0.50 | -0.71 | -0.21 | 0.50 |
| 133 | 0.38 | 0.60 | -1.90 | 0.22 | -2.28 | -2.50 |
| 134 | 1.48 | -1.10 | -2.70 | -2.58 | -4.18 | -1.60 |
| 135 | 0.04 | 0.00 | 1.60 | -0.04 | 1.56 | 1.60 |
| 136 | 0.09 | -2.00 | -1.00 | -2.09 | -1.09 | 1.00 |
| 137 | 0.33 | -2.20 | -1.20 | -2.53 | -1.53 | 1.00 |
| 138 | -0.43 | 0.30 | -0.30 | 0.73 | 0.13 | -0.60 |
| 139 | -0.59 | -1.50 | -0.20 | -0.91 | 0.39 | 1.30 |
| 140 | 0.77 | -0.23 | 1.10 | -1.00 | 0.33 | 1.33 |
| 141 | 0.19 | -3.20 | -1.20 | -3.39 | -1.39 | 2.00 |
| 142 | -0.28 | 1.00 | 0.00 | 1.28 | 0.28 | -1.00 |
| 143 | 0.10 | -0.90 | 1.10 | -1.00 | 1.00 | 2.00 |
| 144 | -0.41 | -0.30 | -1.20 | 0.11 | -0.79 | -0.90 |
| 145 | -0.57 | -2.50 | -1.00 | -1.93 | -0.43 | 1.50 |
| 146 | -0.26 | -1.50 | -2.40 | -1.24 | -2.14 | -0.90 |
| 147 | 1.22 | 1.90 | 0.70 | 0.68 | -0.52 | -1.20 |
| 148 | -0.06 | -0.40 | -1.00 | -0.34 | -0.94 | -0.60 |
| 149 | -0.03 | -0.50 | 1.20 | -0.47 | 1.23 | 1.70 |
| 150 | -0.32 | 0.10 | 1.20 | 0.42 | 1.52 | 1.10 |
| 151 | 0.26 | -0.30 | 0.30 | -0.56 | 0.04 | 0.60 |
| 152 | 0.35 | 0.20 | -0.50 | -0.15 | -0.85 | -0.70 |
| **ID** | **MB-TSB (mg/dL)** | **MB-TcB (Forehead)**  **(mg/dL)** | **MB-TcB (Sternum)**  **(mg/dL)** | **TSB-TcB (Forehead)**  **(mg/dL)** | **TSB-TcB (Sternum)**  **(mg/dL)** | **TcB (Forehead)-TcB (Sternum) (mg/dL)** |
| 153 | -0.40 | -2.70 | -2.30 | -2.30 | -1.90 | 0.40 |
| 154 | -0.54 | -3.90 | -1.30 | -3.36 | -0.76 | 2.60 |
| 155 | -1.10 | -1.40 | -1.20 | -0.30 | -0.10 | 0.20 |
| 156 | 0.63 | -0.60 | 0.50 | -1.23 | -0.13 | 1.10 |
| 157 | 0.45 | 0.60 | 0.80 | 0.15 | 0.35 | 0.20 |
| 158 | -0.72 | -1.70 | -1.80 | -0.98 | -1.08 | -0.10 |
| 159 | 1.25 | 1.80 | 2.90 | 0.55 | 1.65 | 1.10 |
| 160 | 0.51 | -0.30 | 0.30 | -0.81 | -0.21 | 0.60 |
| 161 | -0.35 | -0.10 | -0.70 | 0.25 | -0.35 | -0.60 |
| 162 | -0.30 | -0.50 | -1.50 | -0.20 | -1.20 | -1.00 |
| 163 | -0.15 | -0.70 | 0.70 | -0.55 | 0.85 | 1.40 |
| 164 | -1.36 | -2.30 | -1.80 | -0.94 | -0.44 | 0.50 |
| 165 | -0.51 | -0.10 | -0.20 | 0.41 | 0.31 | -0.10 |
| 166 | -0.21 | -2.00 | -1.20 | -1.79 | -0.99 | 0.80 |
| 167 | 0.61 | 1.00 | 0.20 | 0.39 | -0.41 | -0.80 |
| 168 | 0.47 | 1.50 | -1.00 | 1.03 | -1.47 | -2.50 |
| 169 | -0.08 | -1.20 | -1.80 | -1.12 | -1.72 | -0.60 |
| 170 | -0.50 | -2.40 | -0.80 | -1.90 | -0.30 | 1.60 |
| 171 | 0.13 | -1.70 | -0.90 | -1.83 | -1.03 | 0.80 |
| 172 | 0.29 | -1.40 | -1.90 | -1.69 | -2.19 | -0.50 |
| 173 | -0.08 | -1.90 | -0.20 | -1.82 | -0.12 | 1.70 |
| 174 | -0.33 | -2.80 | -2.80 | -2.47 | -2.47 | 0.00 |
| 175 | 0.39 | -2.40 | -1.50 | -2.79 | -1.89 | 0.90 |
| 176 | -0.93 | -3.10 | -3.10 | -2.17 | -2.17 | 0.00 |
| 177 | -0.11 | -3.00 | -0.60 | -2.89 | -0.49 | 2.40 |
| 178 | 1.21 | -1.80 | -0.80 | -3.01 | -2.01 | 1.00 |
| 179 | 0.37 | 0.30 | -0.80 | -0.07 | -1.17 | -1.10 |
| 180 | 0.45 | 2.00 | 0.80 | 1.55 | 0.35 | -1.20 |
| 181 | 0.28 | -1.60 | 0.10 | -1.88 | -0.18 | 1.70 |
| 182 | 0.14 | -1.60 | -2.20 | -1.74 | -2.34 | -0.60 |
| 183 | 0.12 | -0.80 | -3.20 | -0.92 | -3.32 | -2.40 |
| 184 | -0.05 | 0.00 | -0.70 | 0.05 | -0.65 | -0.70 |
| 185 | 0.48 | -2.50 | -0.70 | -2.98 | -1.18 | 1.80 |
| 186 | -0.06 | -3.10 | -1.10 | -3.04 | -1.04 | 2.00 |
| 187 | -0.70 | -2.10 | -1.70 | -1.40 | -1.00 | 0.40 |
| 188 | -0.23 | 0.40 | -0.60 | 0.63 | -0.37 | -1.00 |
| 189 | -0.33 | -0.90 | -1.90 | -0.57 | -1.57 | -1.00 |
| 190 | -0.39 | -0.30 | -1.10 | 0.09 | -0.71 | -0.80 |
| 191 | 0.23 | -0.20 | 0.60 | -0.43 | 0.37 | 0.80 |
| **ID** | **MB-TSB (mg/dL)** | **MB-TcB (Forehead)**  **(mg/dL)** | **MB-TcB (Sternum)**  **(mg/dL)** | **TSB-TcB (Forehead)**  **(mg/dL)** | **TSB-TcB (Sternum)**  **(mg/dL)** | **TcB (Forehead)-TcB (Sternum) (mg/dL)** |
| 192 | 0.46 | -2.50 | -1.90 | -2.96 | -2.36 | 0.60 |
| 193 | 0.06 | -3.50 | -2.80 | -3.56 | -2.86 | 0.70 |
| 194 | -0.68 | -1.70 | -1.30 | -1.02 | -0.62 | 0.40 |
| 195 | 0.60 | -1.70 | -3.30 | -2.30 | -3.90 | -1.60 |
| 196 | -0.23 | -1.70 | -0.80 | -1.47 | -0.57 | 0.90 |
| 197 | 0.28 | -2.30 | -2.20 | -2.58 | -2.48 | 0.10 |
| 198 | -0.45 | -2.40 | -1.90 | -1.95 | -1.45 | 0.50 |
| 199 | -0.37 | -2.60 | -3.30 | -2.23 | -2.93 | -0.70 |
| 200 | 0.60 | -0.90 | -0.70 | -1.50 | -1.30 | 0.20 |
| 201 | 0.36 | -2.30 | -1.60 | -2.66 | -1.96 | 0.70 |
| 202 | -0.31 | -3.20 | -2.70 | -2.89 | -2.39 | 0.50 |
| 203 | 0.30 | 1.10 | -0.30 | 0.80 | -0.60 | -1.40 |
| 204 | 0.44 | -0.70 | -0.20 | -1.14 | -0.64 | 0.50 |
| 205 | 0.50 | -3.50 | -0.90 | -4.00 | -1.40 | 2.60 |
| 206 | 2.27 | 3.30 | 1.90 | 1.03 | -0.37 | -1.40 |
| 207 | 0.45 | 1.70 | 0.50 | 1.25 | 0.05 | -1.20 |
| 208 | -1.10 | 2.30 | -2.70 | 3.40 | -1.60 | -5.00 |
| 209 | -2.88 | 1.40 | -0.60 | 4.28 | 2.28 | -2.00 |
| 210 | 0.70 | -0.50 | -2.80 | -1.20 | -3.50 | -2.30 |
| 211 | -1.73 | 1.70 | 0.20 | 3.43 | 1.93 | -1.50 |
| 212 | -1.16 | -1.30 | -2.70 | -0.14 | -1.54 | -1.40 |
| 213 | -0.40 | -1.70 | -1.10 | -1.30 | -0.70 | 0.60 |
| 214 | -0.16 | -0.60 | -2.00 | -0.44 | -1.84 | -1.40 |
| 215 | 0.43 | -3.00 | -1.20 | -3.43 | -1.63 | 1.80 |
| 216 | 1.01 | 0.10 | -0.30 | -0.91 | -1.31 | -0.40 |
| 217 | -2.07 | -3.40 | -4.20 | -1.33 | -2.13 | -0.80 |
| 218 | -1.42 | -1.60 | -2.80 | -0.18 | -1.38 | -1.20 |
| 219 | -2.34 | -3.50 | -2.30 | -1.16 | 0.04 | 1.20 |

**Abbreviations:** dL: deciliter; mg: milligram; MB: microbilirubin; TcB: transcutaneous bilirubin; TSB: total serum bilirubin**.** All values reported in mg/dL
